# Generation of CSF1-Independent Ramified Microglia-Like Cells from Leptomeninges In Vitro

**DOI:** 10.3390/cells10010024

**Published:** 2020-12-25

**Authors:** Junya Tanaka, Hisaaki Takahashi, Hajime Yano, Hiroshi Nakanishi

**Affiliations:** 1 Department of Molecular and Cellular Physiology, Ehime University Graduate School of Medicine, Toon, Ehime 791-0295, Japan; h-takahashi@hokuriku-u.ac.jp (H.T.); hajime-y@m.ehime-u.ac.jp (H.Y.); 2 Division of Pathophysiology, Faculty of Pharmaceutical Sciences, Hokuriku University, Kanazawa, Ishikawa 920-1181, Japan; 3 Department of Pharmacology, Faculty of Pharmacy, Yasuda Women′s University, Yasuhigashi, Hiroshima 731-0153, Japan

**Keywords:** CD68, CD163, colony-stimulating factor 1, epidermal growth factor, Iba1, leptomeninges, macrophages, microglia

## Abstract

Although del Río-Hortega originally reported that leptomeningeal cells are the source of ramified microglia in the developing brain, recent views do not seem to pay much attention to this notion. In this study, in vitro experiments were conducted to determine whether leptomeninges generate ramified microglia. The leptomeninges of neonatal rats containing Iba1^+^ macrophages were peeled off the brain surface. Leptomeningeal macrophages strongly expressed CD68 and CD163, but microglia in the brain parenchyma did not. Leptomeningeal macrophages expressed epidermal growth factor receptor (EGFR) as revealed by RT-PCR and immunohistochemical staining. Cells obtained from the peeled-off leptomeninges were cultured in a serum-free medium containing EGF, resulting in the formation of large cell aggregates in which many proliferating macrophages were present. In contrast, colony-stimulating factor 1 (CSF1) did not enhance the generation of Iba1^+^ cells from the leptomeningeal culture. The cell aggregates generated ramified Iba1^+^ cells in the presence of serum, which express CD68 and CD163 at much lower levels than primary microglia isolated from a mixed glial culture. Therefore, the leptomeningeal-derived cells resembled parenchymal microglia better than primary microglia. This study suggests that microglial progenitors expressing EGFR reside in the leptomeninges and that there is a population of microglia-like cells that grow independently of CSF1.

## 1. Introduction

The Spanish scientist who first identified microglia, del Río-Hortega, noted the presence of microglia in early development and proposed that they might initially arise from mesodermal cells of the innermost layer of the meninges, the pia mater. He reported the migration of embryonic corpuscles from the pia into the nerve centers, but simultaneously proposed that microglia may eventually arise from other related elements, mainly blood-derived monocytes, based on their similarities in morphology and phagocytic activity [1]. These two statements have provoked debate on the origin of microglia in the central nervous system (CNS) for decades. However, one of his concepts that blood-derived monocytes are microglial precursors has been largely revised by recent several fate-mapping studies. Microglia originate solely from yolk sac primitive macrophages, which influx into the developing parenchyma through the circulation [2,3,4,5]. Seeded microglial progenitors persist in the CNS and appear to continue to persist until adulthood [6]. The resident microglia are maintained with minimal contribution from circulating monocytes and are internally maintained by self-renewal [2,7,8]. On the other hand, non-parenchymal CNS macrophages found in the meninges, perivascular spaces and choroid plexus were believed to originate from short-living blood monocytes after birth that are quickly replaced by bone marrow-derived cells [9,10]. However, recent genetic fate-mapping approaches combined with parabiosis have also revealed that CNS macrophages arise from yolk sac precursors during embryonic development and remain a stable population [11].

Because of recent advances in the understanding of the origins of microglia and CNS macrophages, little attention has been paid to the other concept of microglial origin proposed by del Rio-Hortega that microglia might initially arise from the pia mater. Leptomeninges containing the pia mater have been carefully removed from immature rat or murine brains before preparing the mixed glial culture for the isolation of primary microglia [12,13]. This may be evidence of the limited acceptance of del Río-Hortega’s original idea. However, little is known about the growth and differentiation of primary leptomeningeal cells in serum-free medium. Here we have shown that Iba1^+^ cells, which are distinct from leptomeningeal macrophages, are generated from the dissociated culture of the leptomeninges. Rather surprisingly, these Iba1^+^ cells resemble parenchymal microglia better than the primary microglia and independent of colony-stimulating factor 1 (CSF1).

## 2. Materials and Methods

### 2.1. Immunohistochemical Staining of Neonatal Rat Brains

Neonatal Wistar rats were used for leptomeningeal isolation and immunohistochemical analysis of their brains and primary mixed glial culture. The animals were handled in accordance with the Guidelines for Animal Experimentation of the Ehime University School of Medicine. For immunohistochemistry, neonatal rats (*n* = 3) were deeply anesthetized and transcardially perfused with a fixative containing 4% paraformaldehyde and 2 mM MgCl_2_ in phosphate-buffered saline (PBS). The dissected brains were immersed in 15% sucrose in PBS at 4 °C overnight, rapidly frozen in powdered dry ice and sliced at the caudoputamen level into 4-µm thick coronal sections using a cryostat [14]. The primary antibodies listed in Table 1 were used for the immunohistochemical staining of the sections. The immunoreaction was visualized with fluorescein isothiocyanate (FITC)- or Cy3-labeled anti-mouse or rabbit IgG secondary antibodies (Chemicon, Temecula, CA, USA). Hoechst 33,258 (Sigma-Aldrich, St Louis, MO, USA) was used for nuclear staining. The immunostained specimens were observed under a BX-52 Olympus (Tokyo, Japan) microscope equipped with a charge-coupled device (CCD) camera and differential interference contrast (DIC) optics.

### 2.2. Culture of Leptomeningeal Cells

After incising the periphery of the cerebral cortices of a neonatal rat using scissors, two pieces of leptomeninges were peeled off both hemispheres of the brain. Careful attention was paid to ensure that the brain tissue was not contaminated. Two culture methods for isolated leptomeninges were employed: (1) Dissociated culture. Pieces of leptomeninges were incubated in trypsin-ethylenediamine tetraacetic acid (EDTA, Sigma-Aldrich) for 20 min at the room temperature with gentle shaking. The leptomeninges were passed through a nylon mesh bag (pore size: 160 µm) using fire-polished Pasteur pipettes, to remove undigested aggregates, and centrifuged. The cells were then resuspended in a serum-free medium (E2) (serum-free Dulbecco’s modified Eagle’s medium (DMEM) with 1 mg/mL glucose (Wako, Osaka, Japan) containing 10 mM 4-(2-hydroxyethyl)-1-piperazineethanesulfonic acid (HEPES; pH 7.3; Roche Diagnostics Gmbh, Mannheim, Germany), 4.5 mg/mL glucose, 5 µg/mL insulin, 5 nM sodium selenite, 5 µg/mL transferrin (Gibco, Grand Island, NY, USA), and 0.2 mg/mL bovine serum albumin (Sigma-Aldrich) containing 10 ng/mL murine recombinant epidermal growth factor (EGF; PeproTech, London, UK), designated E2-EGF, and cultured in polystyrene dishes for suspension culture (Sumitomo, Tokyo, Japan). Dissociated cells cultured in E2-EGF started to form aggregates from the following day of culture. On the 4th or 6th day in vitro, the aggregates and scattered cells on the dishes were collected using a scraper. The collected cells were seeded onto poly-L-lysine (PLL; Sigma-Aldrich)-coated glass coverslips and cultured for 2 days in DMEM containing 10% fetal calf serum (FCS-DMEM). (2) Ex vivo culture. Without trypsinization, pieces of leptomeninges were cultured in E2-EGF on PLL-coated glass coverslips. The pieces gradually became rounder and larger with culture while producing many cells, including Iba1^+^ macrophage-like cells.

### 2.3. Immunocytochemistry of Microglia-Like Cells from Leptomeningeal Cultures

To morphologically examine the nature of the cells generated from leptomeningeal cultures, cells on PLL-coated glass coverslips were fixed in a fixative containing 4% paraformaldehyde and 2 mM MgCl_2_ in PBS. They were then immunostained with the primary antibodies listed in Table 1. As secondary antibodies, anti-mouse or anti-rabbit IgG antibodies labeled with FITC or Cy3 (Chemicon) were used. In some experiments, the cell aggregates and enlarged pieces of the leptomeninges were sectioned into 4-µm thick slices; the sections were then fixed and immunostained.

### 2.4. Preparation of Primary Microglia and Astrocytes from Mixed Glial Culture

Mixed glial cell cultures were started from forebrain cells of newborn Wistar rats after the leptomeninges were completely removed, as described previously [15]. For immunocytochemical study, purified microglial cells were seeded on PLL-coated glass coverslips in FCS-DMEM. Two days after seeding, microglial cells were fixed and immunostained with antibodies against Iba1, CD68 or CD163. For RT-PCR experiments, purified astrocytes or microglial cells seeded on dishes were harvested to obtain total RNA using Isogen (Nippon Gene, Tokyo, Japan) as described elsewhere [16].

### 2.5. Immunoblot Analysis of the Generation of GFAP^+^ or Iba1^+^ Cells

To evaluate the generation of glial fibrillary acidic protein (GFAP)^+^ and Iba1^+^ cells from the culture of leptomeningeal cells, immunoblot analysis was employed as described elsewhere [15]. In brief, cell aggregates were cultured for 6 days in 3.5-cm suspension culture dishes using E2 alone or E2 containing 10 ng/mL EGF, 10 ng/mL basic fibroblast growth factor (bFGF; PeproTech), 20 ng/mL CSF1 (PeproTech), or 20 ng/mL granulocyte/macrophage colony-stimulating factor (GM-CSF; PeproTech). The cells were then transferred onto 4-well plates coated with PLL and cultured for 2 days in FCS-DMEM. The cells were finally solubilized in Laemmli’s sample solution containing sodium dodecyl sulfate (SDS). Immunoreactive bands were visualized using alkaline phosphatase-labeled secondary antibodies (Promega, Madison, WI, USA).

### 2.6. RT-PCR Analysis

Total RNA was extracted from homogenates of the leptomeninges isolated from 8 neonatal rat brains, cultured primary microglia, or astrocytes and reverse-transcribed to obtain single-stranded cDNA, as described elsewhere [16]. The sequences of the primers used were as follows: β-actin as internal standard (primers: sense, AGA AGA GCT ATG AGC TGC CTG ACG; antisense, TAC TTG CGC TCA GGA GGA GCA ATG), epidermal growth factor receptor (EGFR) (primers: sense, TAC CAG CAG GAC TTC TTT CC; antisense, AAC TTC CCA AAG AGC ATC AG) and CSF1 receptor (CSF1R) (primers: sense, CGA GTC AAC AGA GCA ACC AA; antisense, GTG GCT TTT GGG AAG CAG TA). The annealing temperature was 56 °C, and amplification cycle numbers were 22 for β-actin and 30 for EGFR.

## 3. Results

**Figure 1:**
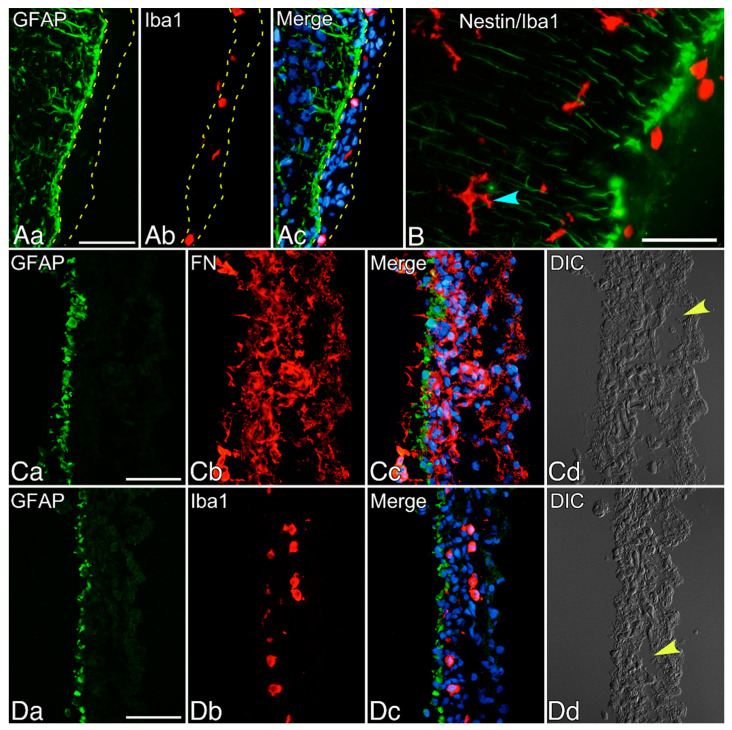
Leptomeninges enveloping the surface of the neonatal rat cerebral cortex. (**A**,**B**) show leptomeninges in Scheme 1. macrophages (red). Nuclei were stained blue by Hoechst 33258. (**B**) GFAP^+^ cells over the surface of the brain parenchyma expressed nestin (green), and Iba1^+^ ramified microglia (red, denoted by a blue arrowhead) were present in the parenchyma. (**C**,**D**) show sections of fixed leptomeninges that were peeled off the brain surface. (**C**) The peeled-off leptomeninges contained a GFAP^+^ monolayer (green) located on the brain surface, and fibronectin^+^ cells (FN, red). Nuclei were stained blue by Hoechst 33258. (**D**) Many macrophages (Iba1, red) were present in the peeled-off leptomeninges contained a GFAP^+^ monolayer (green). Nuclei were stained blue by Hoechst 33258. The yellow arrowheads in (**C**,**D**) denote the subarachnoid space. DIC: differential interference contrast. Scale bars = 50 µm.

The pia and arachnoid mater adhere to each other through arachnoid trabeculae, constituting leptomeninges. The leptomeninges enveloping the surface of the neonatal rat brain contained Iba1^+^ macrophages (Figure 1A). Just under the pia mater, GFAP^+^ cells formed a monolayer over the surface of the cortex. These GFAP^+^ cells expressed nestin and extended straight processes deeply into the parenchyma in the vertical direction (Figure 1B). In the parenchyma, Iba1^+^ ramified microglia were present.

Leptomeninges were isolated after incising the periphery of the cerebral cortex, taking care not to contaminate the brain tissue. The isolated leptomeninges were fixed and perpendicularly sectioned. The sections contained a monolayer of GFAP^+^ cells and scattered macrophages (Figure 1C,D). Most cells in the leptomeninges strongly expressed fibronectin, which may be attributable to the fibroblasts comprising loose connective tissues of the leptomeninges (Figure 1C). The subarachnoid space was identified in the peeled-off leptomeninges by observation with DIC optics.

**Figure 2:**
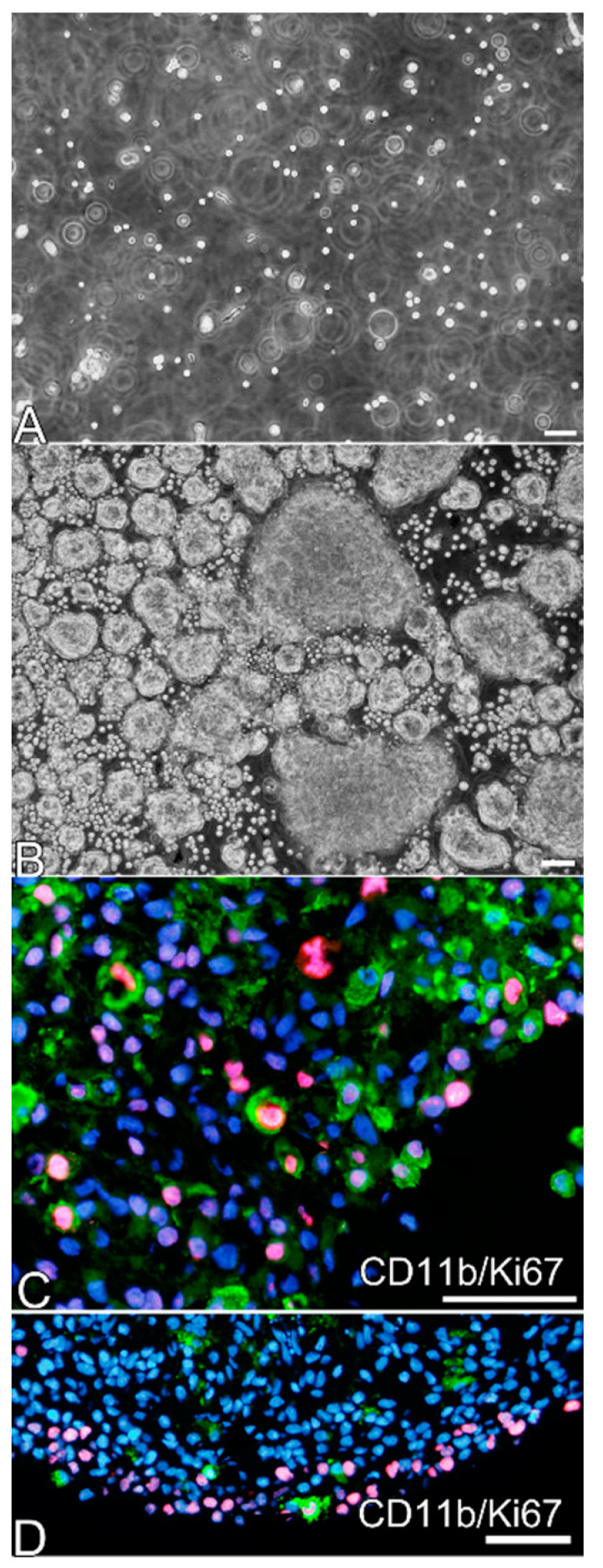
Dissociated and ex vivo cultures of peeled-off leptomeninges. (**A**) A phase contrast micrograph shows isolated cells suspended in E2-epidermal growth factor (EGF) that were obtained from peeled-off leptomeninges of neonatal rat brains by incubating with trypsin-ethylenediamine tetraacetic acid (EDTA). (**B**) After the dissociated cells were cultured for 4 days, they formed large aggregates. (**C**) After fixation, the aggregates shown in (**B**) were sectioned and immunostained with antibodies against CD11b (green), another marker of microglia/macrophages, and Ki67 (red). Many CD11b^+^ amoeboid cells expressed Ki67 in the aggregates. Nuclei were stained blue by Hoechst 33258. (**D**) Sections of an ex vivo culture of leptomeninges maintained in E2-EGF for 6 days. There are also CD11b^+^ (green)/Ki67^+^ (red) cells in an enlarged round piece of leptomeninges. Nuclei were stained blue by Hoechst 33258. Scale bar = 50 µm.

**Figure 3:**
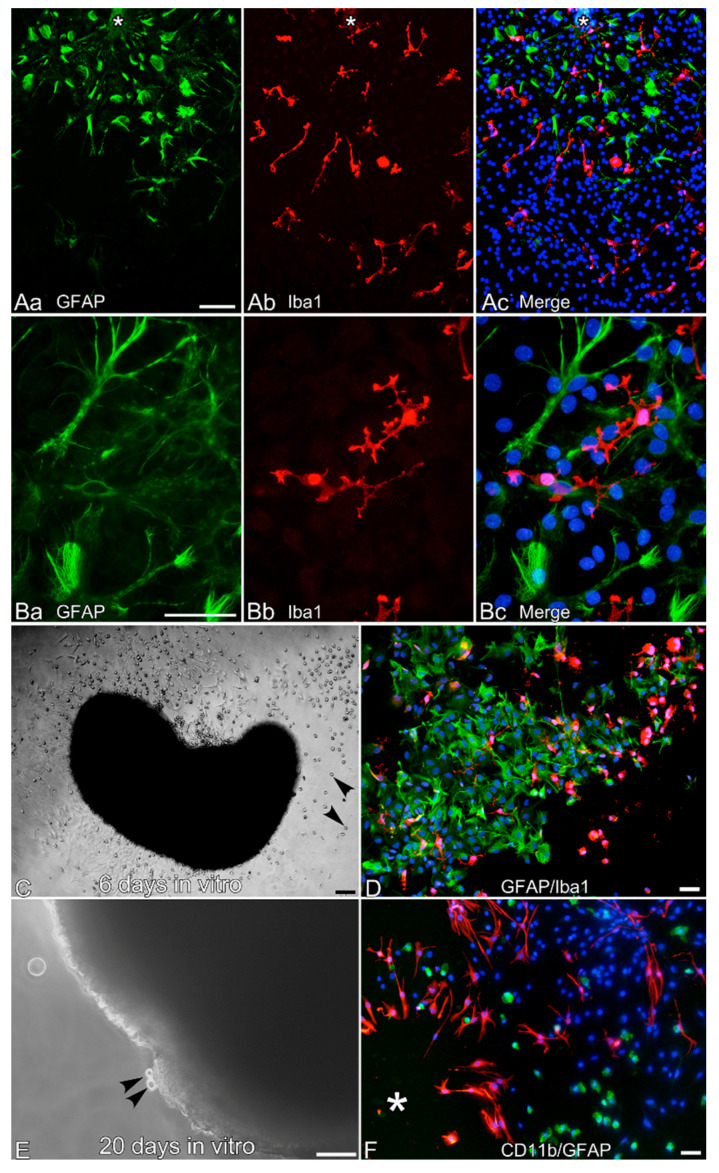
Generation of Iba1^+^ ramified microglia-like cells from leptomeningeal cells on poly-L-lysine (PLL)-coated glass coverslips in Dulbecco’s modified Eagle′s medium containing 10% fetal calf serum (FCS-DMEM). (**A**,**B**) GFAP^+^ cells (green) and Iba1^+^ ramified microglia-like cells (red) generated from aggregates in dissociated culture maintained in E2-EGF for 4 days. The cells were fixed and immunostained after the aggregates were cultured for 2 days in FCS-DMEM. Nuclei were stained blue by Hoechst 33258. Asterisks in (**A**) denote the place where an aggregate loosely attached before fixation. The aggregates detached during fixation, and GFAP^+^ and Iba1^+^ cells migrated out of the aggregates. (**C**,**D**) En bloc culture of leptomeninges-generated Iba1^+^ cells as well as GFAP^+^ cells. (**C**) A phase contrast micrograph shows that a large number of cells migrated out of a rounded piece of meninge cultured for 6 days in E2-EGF. Many amoeboid cells (arrowheads) came out and migrated a long distance. (**D**) Ramified, amoeboid Iba1^+^ cells (red) and GFAP^+^ cells (green) generated from the rounded leptomeninges. Nuclei were stained blue by Hoechst 33258. (**E**) A piece of leptomeninges cultured for 20 days in E2-EGF still produced macrophage-like cells budding from the surface of the leptomeninges (arrowheads). (**F**) Cells derived from the cultured tissue were fixed and immunostained with antibodies against CD11b (green) and GFAP (red). CD11b^+^ cells (green) showed only amoeboid morphology. Nuclei were stained blue by Hoechst 33258. Scale bar = 25 µm (**A**,**D**), 50 µm (**B**,**C**). Generated cells decreased in number with longer culture.

Isolated leptomeninges with a monolayer of GFAP^+^ cells were cultured in dishes for suspension culture using serum-free medium containing EGF (E2-EGF). In the dissociated culture, isolated single cells obtained by treating the peeled-off leptomeninges with trypsin-EDTA were maintained in E2-EGF during the first day of culture (Figure 2A). The cells increased in number and formed large aggregates four days after starting the culture (Figure 2B). Macrophages were scattered inside the aggregates, and many of them expressed Ki67 in their nuclei, implying that they were proliferating (Figure 2C,D). When untrypsinized leptomeninges were cultured in the same way as the ex vivo culture, the pieces of the leptomeninges gradually grew in size and became round (see Figure 3). Inside the growing pieces of leptomeninges were proliferating macrophages (observed through the Ki67 immunohistochemical staining of the sectioned pieces). Other types of cells, including GFAP^+^ cells, were often noted to be Ki67^+^ (data not shown). Thus, the aggregate formation may be attributable not only to cell–cell attachment, but also to cell proliferation.

Leptomeningeal cell aggregates formed in dissociated culture were transferred onto PLL-coated glass coverslips and cultured in DMEM containing 10% FCS (FCS-DMEM). Immediately after the transfer, the aggregates weakly attached to the coverslips and many cells migrated out of the aggregates. The cells that migrated were fixed two days later and immunostained with antibodies to GFAP and Iba1 (Figure 3). Although most cells were GFAP^−^/Iba1^−^, a significant number of Iba1^+^ ramified microglia-like cells and GFAP^+^ stellate cells were present among them (Figure 3A). Ramified microglia-like cells are often located in the vicinity of GFAP^+^ cells, but some ramified microglia-like cells made no direct contact with GFAP^+^ cells. The ramified morphology was more apparent under high magnification (Figure 3B). When leptomeninges in ex vivo culture were transferred onto PLL-coated glass coverslips, many cells migrated out of the rounded leptomeninges. Such a culture, however, produced much more amoeboid cells rather than ramified cells (arrowheads, Figure 3C). Generated cells decreased in number with longer culture.

After 10 days of ex vivo culture, ramified microglia-like cells were no longer generated, although a number of amoeboid macrophages and GFAP^+^ cells were continuously produced even 20 days after the start of culture in E2-EGF (Figure 3E,F). Note that amoeboid CD11b^+^ cells budded out of the rounded leptomeninges, as shown in Figure 3E (arrowheads).

**Figure 4:**
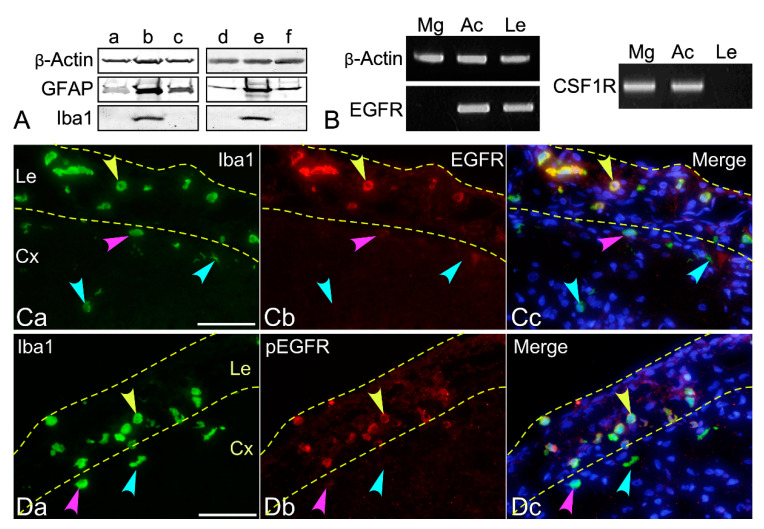
Effects of cytokines or growth factors on the generation of GFAP^+^ and Iba1^+^ cells as revealed by immunoblotting (**A**), and the expression of EGF receptor (EGFR) by leptomeninges (**B**–**D**). Leptomeninges are surrounded by dotted lines in (**C**,**D**). (**A**) Two separate dissociated leptomeningeal cultures from neonatal rats of different dams (a–c and d–f) were maintained for 6 days in serum-free medium (E2) alone (a), or E2 containing EGF (b and e), colony-stimulating factor 1 (CSF1) (c), basic fibroblast growth factor (bFGF) (d), or granulocyte macrophage colony-stimulating factor (GM-CSF) (f), while they were forming cell aggregates. The aggregates were then cultured in FCS-DMEM for 2 days, followed by immunoblotting using antibodies against β-actin, GFAP, or Iba1. Iba1 immunoreactivity was observed only when the cells were cultured in the presence of EGF. GFAP immunoreactivity was most enhanced in the presence of EGF. (**B**) mRNA encoding EGFR was expressed by primary cultured astrocytes (Ac) and freshly isolated leptomeninges of the neonatal rat brains (Le), while primary microglia (Mg) did not express EGFR mRNA, as revealed by RT-PCR. mRNA encoding CSF1R was expressed by Mg and Ac, while Le did not express CSF1R mRNA. (**C**) EGFR expression by Iba1^+^ macrophages (green, denoted by a yellow arrowhead) in the leptomeninges (Le) of the neonatal rat brain was immunohistochemically detected. Amoeboid microglia (green, denoted by a pink arrowhead) in the cerebral cortex (Cx) weakly expressed EGFR (red). Amoeboid microglia in the deeper layer and ramified microglia (denoted by blue arrowheads) did not express EGFR. Astrocytes were EGFR^-^. Nuclei were stained blue by Hoechst 33258. (**D**) Immunoreactivity of phosphorylated EGFR (pEGFR, red) was also detected in macrophages in the leptomeninges (green, denoted by a yellow arrowhead), but not in most microglia in the parenchyma (green, denoted by a blue arrowhead). A few amoeboid microglia in the cortex (green, denoted by a pink arrowhead) weakly expressed pEGFR (red). Scale bar = 50 µm.

The results shown above were obtained using leptomeningeal cells cultured in E2-EGF. The effect of EGF on the generation of ramified and amoeboid macrophages was investigated by immunoblotting (Figure 4A). Two separate dissociated leptomeningeal cultures from neonatal rats of different dams were maintained for six days in the absence of cytokines (Figure 4A), and in the presence of EGF (Figure 4B,E), CSF1 (Figure 4C), bFGF (Figure 4D), or GM-CSF (Figure 4F); they were then cultured in FCS-DMEM for two days. EGF was the most effective at generating GFAP^+^ or Iba1^+^ cells among the cytokines and growth factors examined. To see whether leptomeninges, primary astrocytes or microglia express EGFR or not, RT-PCR experiments were performed to detect mRNA encoding EGFR (Figure 4B). As described previously [17], EGFR mRNA expression was not detectable in primary microglia (Mg) (Figure 4B). On the other hand, primary astrocytes (Ac) and the leptomeninges (Le) expressed EGFR mRNA. Immunohistochemical staining using antibodies against EGFR and phosphorylated EGFR (pEGFR) revealed that leptomeningeal macrophages in neonatal rat brains expressed EGFR and that the EGFR expressed was phosphorylated (Figure 4C,D). Amoeboid and ramified microglia did not express or only scarcely expressed EGFR. In contrast to the RT-PCR results, parenchymal astrocytes did not express EGFR. Interestingly, CSF1 had no effect on generating Iba1+ cells (Figure 4A). Furthermore, the leptomeninges did not express CSF1 receptor mRNA (Figure 4B). The results indicate that leptomeningeal macrophages express EGFR, which are activated presumably in response to the endogenous ligand [18].

**Figure 5:**
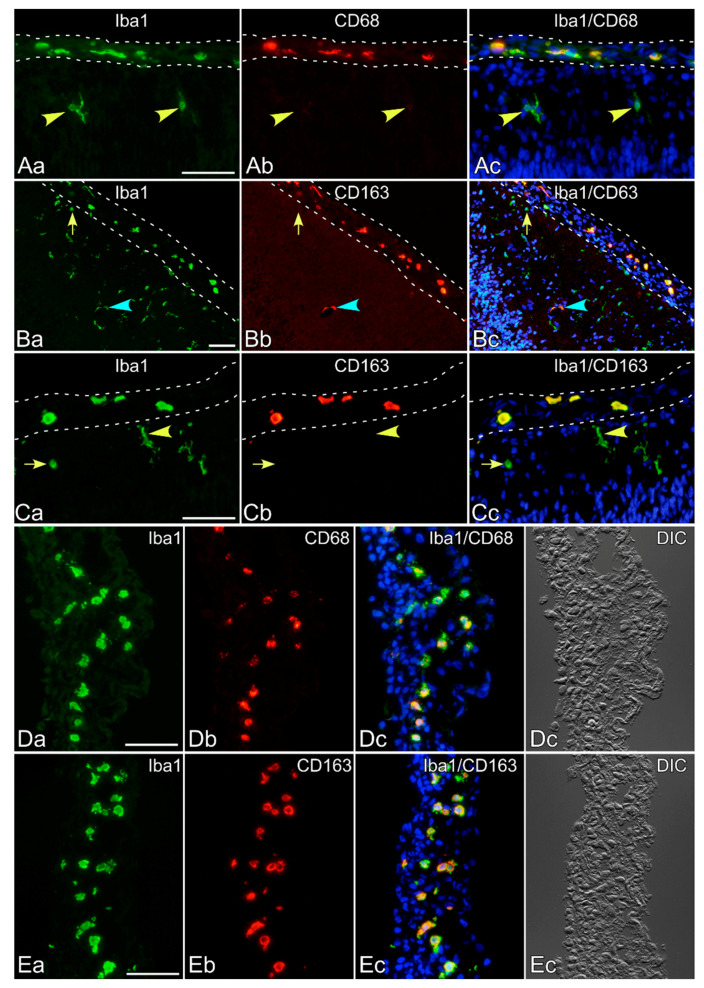
Expression of CD68 and CD163 in neonatal rat brains. (**A**–**C**) Sections of the neonatal rat cerebral cortex were immunostained with antibodies against Iba1, CD68, or CD163. Leptomeninges are surrounded by dotted lines. (**A**) Macrophages (green) in the leptomeninges strongly expressed CD68 (red), whereas ramified microglia in the brain parenchyma (green, denoted by yellow arrowheads) faintly expressed it. Nuclei were stained blue by Hoechst 33258. (**B**,**C**) Macrophages in the leptomeninges, but not ramified microglia in the brain parenchyma (a yellow arrowhead in (**C**)), expressed CD163 (red). Even amoeboid microglia in the parenchyma (denoted by a yellow arrow in (**B**,**C**)) were CD163^−^. Pericytes around small vessels (green, denoted by a blue arrowhead in (**B**)) expressed CD163 (red). Nuclei were stained blue by Hoechst 33258. (**D**,**E**) The peeled-off leptomeninges contained amoeboid cells that expressed CD68 (**D**) and CD163 (**E**). Almost all macrophages (red) expressed both CD markers (red). Nuclei were stained blue by Hoechst 33258. Scale bar = 50 µm.

The monoclonal antibodies ED1 and ED2, which recognize rat CD68 and CD163, respectively, have long been used to identify cells belonging to macrophage/monocyte lineages. CD68, a lysosome-associated membrane protein, is used as a marker for both activated, amoeboid, and primed microglia, but not microglia in the normal healthy brain [19,20]. On the other hand, CD163, the hemoglobin scavenger receptor, enables the distinction between parenchymal microglia and CNS macrophages [21,22]. In fact, the ramified microglia in the neonatal rat brain parenchyma did not express CD68 or expressed it at quite a low level (Figure 5A), whereas macrophages in the leptomeninges strongly expressed it. Thus, ED1 is not suitable to identify ramified microglia in the normal brain parenchyma, as has been demonstrated elsewhere [23,24,25]. Similarly, leptomeningeal macrophages but not microglia expressed CD163 (Figure 5B,C). Iba1^+^ pericytes expressed CD163 (Figure 5B, s blue arrowheads), as has been described elsewhere [23,26]. Thus, microglia in the brain parenchyma were CD68^−^ or weak, and CD163^−^ The isolated leptomeninges contained macrophages expressing CD68 and CD163 (Figure 5D,E).

**Figure 6:**
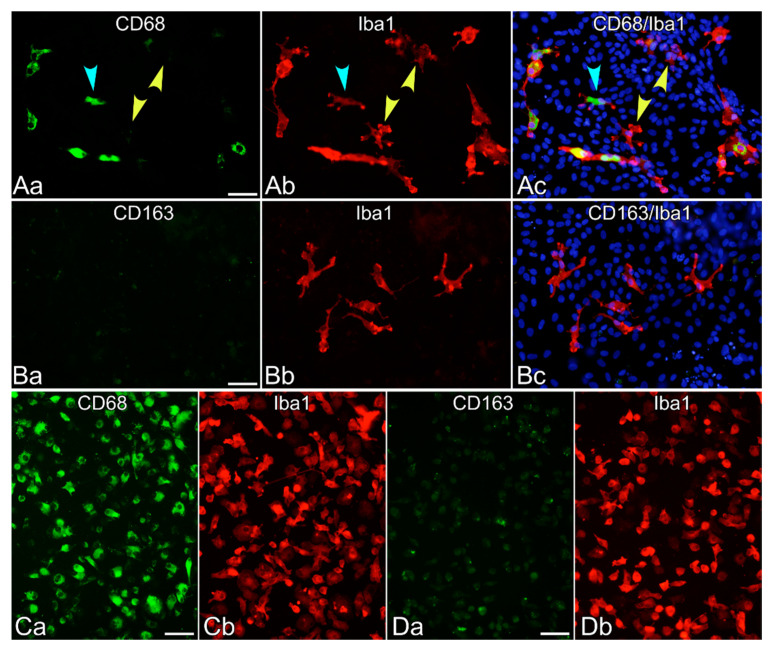
Expression of CD68 and CD163 in Iba1^+^ cells derived from dissociated leptomeningeal aggregates in the dissociated culture (**A**,**B**) or from the mixed glial culture (**C**,**D**). (**A**) Both CD68^+^ (green, denoted by blue arrowheads) and CD68^−^ or weak (green, denoted by yellow arrowheads) cells were observed among Iba1^+^ cells (red) derived from the leptomeningeal culture. Nuclei were stained blue by Hoechst 33258. (**B**) No significant expression of CD163 (green) was observed in any of the leptomeninges-derived Iba1^+^ cells (red). Nuclei were stained blue by Hoechst 33258. (**C**) Almost all primary microglia (Iba1, red) isolated from the mixed glial culture strongly expressed CD68 (green). (**D**) Significant punctate immunostaining of CD163 (green) was observed in many of the primary microglia (Iba1, red). Cells shown here were fixed after they were cultured in FCS-DMEM on PLL-coated glass coverslips for 2 days. Scale bar = 50 µm.

To determine whether Iba1^+^ cells generated from leptomeningeal culture are comparable to microglia in the brain parenchyma in terms of CD68 and CD163 expression, the generated cells were immunostained with the antibodies ED1 and ED2 (Figure 6). Many generated cells from the dissociated culture of the leptomeninges expressed CD68 at quite a low level (Figure 6A), and none of them significantly expressed CD163 (Figure 6B). In contrast, the majority of Iba1^+^ primary microglia isolated from the mixed glial culture, which has traditionally been used as a source of cultured microglia, strongly expressed CD68 (Figure 6C) [13,27]. The punctuate staining of CD163 was also remarkable in the primary microglia (Figure 6D), most of which displayed amoeboid morphology in the presence of serum on PLL-coated glass coverslips.

## 4. Discussion

This study demonstrated that the leptomeningeal macrophages of neonatal rats generated ramified microglia-like cells in vitro. Although leptomeningeal macrophages expressed both CD68 and CD163, ramified cells coming out of leptomeningeal aggregates were CD68^−^ or weak, and all of them were CD163^−^, similar to microglia in the brain parenchyma. On the other hand, the primary microglia isolated from the mixed glial culture were CD68^+^ and CD163^+^. Primary microglia maintain a strong expression of CD68, even when they become ramified in co-culture with astrocytes [13,27,28]. These present observations are consistent with previous studies showing that ramified microglia in the healthy brain parenchyma are generally characterized by a low expression of molecules like CD68 and the major histocompatibility complex II, and by a low phagocytic activity [29,30,31]. The enhancement effects of astrocytes on the ramification of primary microglia appear to be dependent on non-diffusible factors rather than on diffusible ones [13,27,28,32]. However, there were some leptomeninges-derived microglia-like cells that made no direct contact with GFAP^+^ cells (Figure 3A). Thus, spontaneous ramification appeared more pronounced in the leptomeninges-derived cells than in primary microglia from mixed glial culture. Taken together, these results suggest that the leptomeninges-derived microglia-like cells may more closely resemble ramified microglia in the brain than primary microglia from mixed glial cultures.

Leptomeningeal macrophages proliferated in serum-free medium in the presence of EGF. The present immunocytochemical and RT-PCR studies support the notion that they responded directly to EGF through EGFR expressed on their own surfaces. Phosphorylated EGFR in leptomeningeal macrophages was also detected by immunohistochemical examination. On the other hand, microglia in the brain parenchyma and primary microglia did not express EGFR, which is consistent with previous reports [17,33,34]. In fact, primary microglia did not proliferate in E2-EGF.

Osteopetrotic (op/op) mice harbor an inactivating in the coding region of the CSF1 gene and are CSF1 deficient. A significant number of ramified microglial cells were observed in the brain parenchyma of CSF1-deficient op/op mice [35,36,37], although microglia are said to express receptors for CSF1 and GM-CSF [38] and to proliferate in response to both cytokines [39,40]. This study, however, demonstrated that either CSF1 or GM-CSF did not enhance Iba1 expression in leptomeningeal cultures. Considering the present results and the literature, it is likely that the generation of ramified microglia-like cells from the leptomeninges during embryonic development is dependent on ubiquitously produced growth factors such as EGF [18] rather than on hematopoietic cytokines such as CSF1. In fact, we could not detect the expression of CSF1 receptor mRNA in the leptomeninges. The decrease in the number of microglia in the brain of CSF1-deficient op/op mice, however, suggests the presence of a population of microglia that grow dependently on CSF1. More recently, Elmore et al. [41] have found that proliferating cells present three days after the cessation of treatment with PLX3397, an inhibitor of CSF1 receptors, exhibited less complex morphologies than microglia, were Iba1^−^, and expressed markers of stem/progenitor cells, including nestin and CD45. Therefore, they suggested the existence of microglial progenitors. Collectively, there might be two lineages of microglia with different natures in terms of response to growth factors; one responds to EGF, the other to CSF1.

del Río-Hortega argued for the invasion of microglial progenitors through the pial surface [1]. The invasion of the progenitors into the brain parenchyma resembles the budding of macrophages from leptomeningeal aggregates, as shown in Figure 3E [1,42]. However, it remains to be demonstrated in vitro that leptomeningeal macrophages can actually transform into ramified cells. The present study shows that leptomeninges do give rise to ramified Iba1^+^ cells. This in vitro result resembles the in vivo observation made by del Río-Hortega in the early twentieth century [1]. Embryonic amoeboid microglia are reported to migrate along Müller cell end-feet in the quail retina [43,44]. In this study, it is demonstrated that GFAP^+^ cells in the monolayer that firmly attach to the leptomeninges enveloping cerebral cortices extend nestin^+^ long straight processes deeply into the brain parenchyma. Because these GFAP^+^ cells resemble Müller cells in terms of morphology and expressed antigens [45], Iba1^+^ amoeboid cells in the leptomeninges might also invade into the brain parenchyma along the processes of the GFAP^+^ cells.

It may be concluded that progenitors reside in the leptomeninges and serve as one of sources to renew brain CSF1-independent microglia. Further in vivo studies are necessary to elucidate whether the leptomeninges are the progenitor pools for CSF1-independent microglia.

## Figures and Tables

**Table 1:** Primary Antibodies used in this study.

Antigen	Antibody	Dilution	Source
CD11b	Mouse monoclonal (MRC OX-42)	200	Serotec (Oxford, UK)
CD68	Mouse monoclonal (ED1)	200	Serotec (Oxford, UK)
CD163	Mouse monoclonal (ED2)	200	Serotec (Oxford, UK)
EGFR	Mouse monoclonal	200	BD Biosciences (Tokyo, Japan)
pEGFR	Mouse monoclonal (9H2)	200	Santa Cruz (Santa Cruz, CA, USA)
FN	Rabbit polyclonal	500	Sigma-Aldrich (St. Louis, MO, USA)
GFAP	Rabbit polyclonal	500	Shima (Tokyo, Japan)
GFAP	Mouse monoclonal	500	Chemicon (Temecula, CA, USA)
Iba1	Rabbit polyclonal	5000	Wako (Osaka, Japan)
Ki67	Rabbit monoclonal (SP6)	500	NeoMakers (Fremont, CA, USA)

FN: fibronectin; EGFP: epidermal growth factor receptor; pEGFR: phosphorylated EGFR.

## Data Availability

No new data were created or analyzed in this study. Data sharing is not applicable to this article.
